# Platform Trials to Expedite Drug Development in Alzheimer’s Disease: A Report from the EU/US CTAD Task Force

**DOI:** 10.14283/jpad.2021.21

**Published:** 2021-05-24

**Authors:** P. S. Aisen, R. J. Bateman, M. Carrillo, R. Doody, K. Johnson, J. R. Sims, R. Sperling, B. Vellas, EU/US CTAD Task Force

**Affiliations:** 1 Alzheimer’s Therapeutic Research Institute (ATRI), Keck School of Medicine, University of Southern California, San Diego, CA USA; 2 Washington University School of Medicine, St. Louis, MO USA; 3 Alzheimer’s Association, Chicago, IL USA; 4 Genentech/Roche, Basel, Switzerland; 5 Massachusetts General Hospital, Harvard Medical School, Boston, MA USA; 6 Eli Lilly and Company, Indianapolis, IN USA; 7 Brigham and Women’s Hospital, Boston, MA USA; 8 Gerontopole, INSERM U1027, Alzheimer’s Disease Research and Clinical Center, Toulouse University Hospital, Toulouse, France; 9 University of Southern California Alzheimer’s Therapeutic Research Institute, San Diego, CA USA

**Keywords:** Alzheimer’s disease, anti-amyloid therapies, anti-tau therapies, platform trials, adaptive trial design, shared placebos, master protocols, secondary prevention

## Abstract

A diverse range of platforms has been established to increase the efficiency and speed of clinical trials for Alzheimer’s disease (AD). These platforms enable parallel assessment of multiple therapeutics, treatment regimens, or participant groups; use uniform protocols and outcome measures; and may allow treatment arms to be added or dropped based on interim analyses of outcomes. The EU/US CTAD Task Force discussed the lessons learned from the Dominantly Inherited Alzheimer’s Network Trials Unit (DIAN-TU) platform trial and the challenges addressed by other platform trials that have launched or are in the planning stages. The landscape of clinical trial platforms in the AD space includes those testing experimental therapies such as DIAN-TU, platforms designed to test multidomain interventions, and those designed to streamline trial recruitment by building trial-ready cohorts. The heterogeneity of the AD patient population, AD drugs, treatment regimens, and analytical methods complicates the design and execution of platform trials, yet Task Force members concluded that platform trials are essential to advance the search for effective AD treatments, including combination therapies.

## Introduction

**D**isappointing clinical trial results continue to mount in the search for effective treatments for Alzheimer’s disease (AD) despite increasing knowledge about underlying mechanisms and potential therapeutic targets (1, 2). As trials move into earlier stages of the disease with a focus on secondary and even primary prevention, the need for innovative trial designs and improved biomarker technologies has become increasingly evident. In December 2020 as the COVID-19 pandemic caused the suspension of many non-COVID-19 clinical trials worldwide (3), the EU/US Clinical Trials in Alzheimer’s Disease Task Force met virtually to explore platform trials as a means of accelerating and improving the efficiency and success of AD drug development. The Task Force brought together leaders from existing and planned AD platform trials to discuss their experiences to date as well as plans for moving forward. Together, they examined recent experience from the Dominantly Inherited Alzheimer Network Trials Unit (DIAN-TU) platform trial and other platform trials that have launched or are in the planning stages, and that provide lessons applicable to the design of future trials.

Platform trials are those conducted within an infrastructure that enables simultaneous and perpetual assessment of multiple therapeutics, treatment regimens, and/or participant groups. They have the potential to increase efficiency by minimizing screen failures; using uniform outcome measures, protocols, and consents; and testing multiple targets and drugs in parallel rather than serially. Adaptive platform trials may allow new arms to be added or individual arms to be stopped if interim analysis indicates a failure to demonstrate efficacy.

However, platform trials can be highly complex both operationally and analytically, requiring a great deal of coordination to bring multiple stakeholders together at the same time and to produce convincing results.

## Lessons learned from DIAN-TU

**Figure 1:**
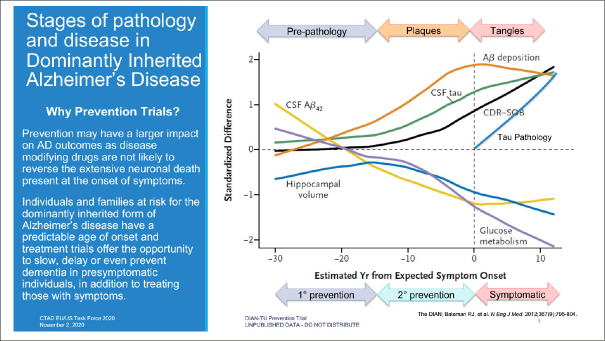
Stages of Pathology and Disease in Dominantly Inherited Alzheimer’s Disease

DIAN-TU and the DIAN Observational study (DIAN-Obs) are public-private partnerships created to facilitate the development of AD therapeutics and advance scientific understanding of the optimal ways to prevent and treat AD. To accomplish these objectives, DIAN enrolled participants from around the world with dominantly inherited AD (DIAD) mutations that confer nearly 100 percent certainty of developing AD. By longitudinally monitoring both symptomatic and presymptomatic participants with clinical assessments and physiologic and pathologic biomarkers, DIAN established the progression of biomarkers across the continuum of the disease and demonstrated a biomarker profile associated with presymptomatic disease (4). This provided a rationale for starting a prevention trial in the presymptomatic stage. Figure 1 illustrates the relationship of a range of biomarkers to disease stage and how that translates to the potential for primary and secondary prevention as well as symptomatic treatment.

Bateman, Randall J., Chengjie Xiong, Tammie L.S. Benzinger, Anne M. Fagan, Alison Goate, Nick C. Fox, Daniel S. Marcus, et al. “Clinical and Biomarker Changes in Dominantly Inherited Alzheimer’s Disease.” New England Journal of Medicine 367, no. 9 (August 30, 2012): 795–804. 10.1056/NEJMoa1202753. Reprinted with permission.

DIAN-TU built a flexible and robust platform based on observational data from DIAN-Obs along with disease progression models, comprehensive biomarker studies, input from participants and family members, and inclusive discussions with stakeholders. The platform enabled the design and execution of prevention studies that can accommodate a range of prevention and treatment trials. Since the rarity of these mutations limits the number of trial participants, DIAN-TU has established study sites around the globe: in North and South American, Europe, Japan, Korea, and Australia, with additional sites planned in China and other countries.

The DIAN-TU Trial Platform is designed to improve the efficiency of trials by:

- testing multiple drugs and targets in parallel
- optimizing the trial design in view of the limited number of participants
- using a pooled placebo and control group, building up data over time to accumulate power to detect a drug effect
- evaluating the magnitude of target engagement based on each drug’s mechanism of action
- adapting the trial in response to biomarker findings, magnitude of drug effects at different doses, and safety signals
- incorporating novel biomarkers
- applying learnings from each study into future studies in the platform.

The DIAN-TU platform launched in 2012 as a 4-year secondary prevention trial in participants with DIAD mutations and expected onset ranging from −15 to +10 years and Clinical Dementia Rating (CDR) score of 0 (cognitively normal), 0.5 (very mild cognitive impairment), or 1 (mild cognitive impairment consistent with early dementia) (6). Nearly 200 participants were enrolled, randomized to receive Eli Lilly’s solanezumab (a soluble anti-amyloid-beta monoclonal antibody), Roche’s gantenerumab (an aggregated anti-amyloid monoclonal antibody), or placebo. A third drug arm testing a betasecretase inhibitor was launched but was terminated early because of safety concerns.

As the two-drug trial continued, adaptations to the platform were made. Planned as a two-year biomarker trial, the study transitioned to a four-year cognitive endpoint trial after the biomarker analysis confirmed that the drugs were engaging their targets. Part way through the trial, a decision was made to increase the dose of both drugs based on external data.

Other components were also added to the platform. These included a run-in period during which data from cognitive measures and tau PET studies were collected in preparation for launching three tau next generation (NexGen) drug arms. Meanwhile, a primary prevention trial in DIAD participants 10 years before symptom onset is in the launch preparation stage. This trial will use a beta amyloid (Aβ)-targeting drug with the aim of preventing the development of plaques before they start.

Topline results for the solanezumab/gantenerumab trial, reported in early 2020, showed that both drugs failed to meet the prespecified primary cognitive endpoint (7). Nonetheless, the platform itself succeeded in meeting many of its objectives and has provided extensive data that continues to be analyzed. While DIAN-TU has not yet confirmed the clinical or cognitive effectiveness of a therapy for DIAD, the ongoing studies have gathered important data relevant to the validity of the amyloid hypothesis, the timing of treatment, and the use of biomarkers to track therapeutic effectiveness.

These analyses suggest two possible reasons that may explain the failure to detect a significant cognitive difference between groups: 1) although the dose was increased part way through the trial, most participants had received lower doses; by the time they were switched to the high dose, many symptomatic participants had already declined substantially, thus limiting the ability to evaluate the effect of the high dose; 2) some asymptomatic trial participants failed to decline and in some cases improved; thus it was not possible to detect a drug effect in those participants.

The continuing data analysis also suggested that gantenerumab showed significant biologic effects on downstream biomarkers, suggesting that higher doses and longer treatment periods may be needed, especially for asymptomatic mutation carriers. While substantially advancing the field, these results were not able to definitively test the hypothesis. Nonetheless, gantenerumab is being studied in an ongoing exploratory open label extension (8).

Other lessons learned from the trial include the need to enrich the study for participants likely to show signs of cognitive decline during the treatment period. The optimal approach to this challenge is not clear, but further analysis of biomarker data from this trial may provide some insight. In addition, other research suggests that some novel biomarkers or combinations of markers may be predictive of incipient cognitive decline. How they would be implemented in screening for trials, however, would present additional complexity. Researchers also continue to search for more sensitive cognitive assessments and tools that minimize practice effects, which could also potentially improve the ability to detect a treatment effect. Yet even if a test is developed to detect subtle cognitive change, the ability of that test to be applied to an AD trial could be limited if the cognitive change results from non-AD aging effects.

## AHEAD 3-45 Platform Study

The AHEAD Study is a platform-based study comprising two Phase 3 secondary prevention trials — the A45 trial and the A3 trial. Both four-year trials are testing Eisai’s anti-Aβ monoclonal antibody lecanemab (BAN 2401) but use a different dosing regimen depending on baseline amyloid burden. Together they are evaluating the efficacy of the treatment across the continuum of early-to-late preclinical AD as determined by positron emission tomography (PET) amyloid assessment. Using different radioligands, PET imaging enables the visualization of both amyloid plaques and tau neurofibrillary tangles. The AHEAD trials build on the findings of the Harvard Aging Brain Study (HABS) showing that early cortical amyloid accumulation is associated with subsequent tau accumulation in the inferior temporal neocortex, which is associated with neurodegeneration and cognitive decline (9). This study and others in separate cohorts have shown that a period of rapid acceleration in rate of Aβ accumulation immediately precedes reaching the threshold of amyloid positivity that heralds cognitive decline (10–12). These studies led to the hypothesis that decreasing amyloid accumulation at the earliest detectable stage will provide the best opportunity to slow progression and prevent cognitive decline.

The A3 trial aims to capture people with a measurable amyloid PET signal that is below a conventional threshold of amyloidosis. These participants with “intermediate” levels of amyloid (early preclinical or “pre-preclinical” AD) will be randomized to receive the drug or placebo monthly. Amyloid PET will be used as the primary outcome measure to determine whether lecanemab slows amyloid accumulation. Tau PET will be a key secondary outcome and cognitive testing will be an exploratory outcome.

In the A45 study, participants with elevated amyloid will be randomized to receive the drug or placebo biweekly for the first two years followed by monthly maintenance dosing, with cognitive testing as the primary outcome. Amyloid and tau PET will be key secondary outcomes, and biomarker studies will also be an essential component of the trial along with additional cognitive and participant-reported outcomes.

The first participants in AHEAD were screened in July and randomized in September. Worldwide, 100 sites have been identified. The AHEAD platform will use a common screening protocol for the two trials, 18F NAV4694 amyloid and 18F MK6240 tau PET at baseline, 2 and 4 years, and will enroll participants between the ages of 55 and 80. Participants under age 65 will need to have one additional risk factor (e.g., family history, APOE carriage, or known amyloid status). In response to the COVID-19 pandemic, the study team also plans to implement home infusion and potentially, supervised remote assessments.

## Tau platforms

Other platforms are currently being planned to evaluate the efficacy of anti-tau drugs. While anti-tau therapies have gained support in the Alzheimer’s community because tau is thought responsible for neurodegeneration and cognitive impairment and because of the disappointing results of so many anti-amyloid therapies, many challenges remain due to the complexity of tau biology and the incomplete understanding of the relationship between tau and Aβ (13, 14). Moreover, several recent trials of anti-tau monoclonal antibodies have failed to demonstrate efficacy, raising concerns that the disappointing search for an anti-amyloid therapy may be repeated for tau therapies (15). Nonetheless, these negative anti-tau trials may in fact highlight even more strongly the importance of a platform approach.

Eli Lilly and Company has proposed using a master protocol, which should facilitate greater efficiency and accelerated drug development through the use of a common protocol, trial design, and infrastructure. Master protocols can be used in umbrella, basket, or adaptive platform trials to evaluate multiple therapies in the context of a single disease, a single therapy in the context of multiple diseases or disease subtypes, or multiple therapies in a single disease with arms added and/or terminated over time (16). For example, Lilly developed an innovative master protocol to evaluate multiple interventions for chronic pain, in which three different therapeutics were tested across three different pain states.

Master protocols offer the opportunity for increased flexibility to explore multiple hypotheses and different drug combinations or patient populations. As with other platform trials they allow for shared placebo data, unbalanced randomization so that more participants are assigned to the active drug arm, and the potential for a lower screen failure rate. While master protocols may offer increased efficiency for testing multiple therapeutics in parallel, tradeoffs include increased complexity, reduced efficiency gains if fewer therapeutics enter as well as diminished flexibility compared to a single therapeutic trial. It is essential to consider the mechanistic differences among candidate tau therapeutics (and among immunotherapies targeting different epitopes) which may point to different selection criteria and outcome measures.

To study treatments for tauopathies, a master protocol could be used to test multiple therapeutics in different tauopathies (e.g. AD, Primary Supranuclear Palsy [PSP], and Corticobasal degeneration [CBD]); multiple therapeutics in one tauopathy at different stages of disease (late stage AD, early symptomatic AD, and preclinical AD); or multiple therapeutics in only one stage (e.g., preclinical AD). Lilly’s consideration of a master protocol approach to testing multiple anti-tau assets could use one or a combination of these structures, although all of them present substantial operational costs and risks.

A second tau platform protocol is being developed by the Alzheimer’s Clinical Trial Consortium (ACTC). Developers of the Alzheimer’s Tau Platform (ATP) envision a proof-of-concept platform to accelerate decision making in tau therapeutic development by simultaneously testing different anti-tau mechanisms in sporadic AD. They argue that a platform that enables testing multiple molecules and multiple regimens using factorial adaptive designs would improve the efficiency of recruitment, trial startup, and analyses. They also suggest that demonstrating efficacy may be possible more quickly with anti-tau versus anti-amyloid therapies since recent evidence suggest that tau PET agents can detect highly dynamic change in some individuals, e.g. those with higher levels of amyloid (17).

DIAN-TU is also planning to add three anti-tau drug arms to its study in 2021. Since platforms can be especially helpful in assessing treatment response at different stages of disease, each arm will likely enroll both presymptomatic and symptomatic participants. Moreover, by testing different mechanisms and targets in the platform and using short-term biomarker studies, the platform approach may be able to get information on which targets are druggable at which stages of disease.

## The landscape of AD platform trials

The concept of platform trials is broad. DIAN-TU was designed to enable testing of multiple drugs using a shared placebo arm, with adaptive allocation based on randomization data. In contrast, the AHEAD 3–45 Platform provides benefits that are primarily operational rather than statistical comparisons between treatment regimens. Platform trials may also be used to explore multifaceted hypotheses, for example, to ask questions such as whether amyloid accumulation must be cleared before targeting tau. Different arms of a platform study can ask different questions simultaneously rather than sequentially to increase efficiency and accelerate drug development.

Longitudinal biomarker and clinical studies in clearly defined cohorts (e.g. APOE homozygotes and heterozygotes) are needed to inform the design of platform programs. A matrix of outcomes (including tau and amyloid biomarkers, imaging, and the neural tool kit) across the continuum of disease could eventually lead to a surrogate marker of efficacy, which could dramatically accelerate the search for effective therapies. For example, the European Prevention of Alzheimer’s Dementia (EPAD) platform began with a longitudinal cohort study to serve as a readiness cohort for proof-of-concept secondary prevention AD trials (18).

Meanwhile, an international network of multidomain intervention clinical trials has been established to replicate the groundbreaking Finnish Geriatric Intervention Study to Prevent Cognitive Impairment and Disability (FINGER), which demonstrated that a combination of lifestyle interventions reduces the risk of cognitive decline in older adults with vascular risk factors that increase their risk of dementia. The Worldwide FINGERS initiative (WW-FINGERS) provides a platform for investigators in different countries and cultures to adapt the FINGERS protocol while using similar protocols and sharing data (19).

The Trial-Ready Cohort for Preclinical/prodromal Alzheimer’s Disease (TRC-PAD) project is a collaborative effort to establish an efficient mechanism for recruiting participants into very early stage Alzheimer’s disease trials (20, 21). Clinically normal and mildly symptomatic individuals are followed longitudinally in a web-based component called the Alzheimer’s Prevention Trial Webstudy (APT Webstudy), with quarterly assessment of cognition and subjective concerns. The Webstudy data is used to predict the likelihood of brain amyloid elevation; individuals at relatively high risk are invited for in-person assessment in the TRC screening phase, during which a cognitive battery is administered and APOE genotype is obtained followed by reassessment of risk of amyloid elevation (22–24). After an initial validation study, plasma amyloid peptide ratios will be included in this risk assessment. Based on this second risk calculation, individuals may have amyloid testing by PET scan or lumbar puncture, with those potentially eligible for trials followed in the TRC, while the rest are invited to remain in the APT Webstudy.

## Addressing the challenges of AD platform trials

The heterogeneity of the AD patient population creates several complications in designing and executing platform trials. For example, the substantial clinical and biomarker heterogeneity in sporadic AD makes screening particularly challenging and increases the sample size needed to obtain adequate power. Regarding screening, accumulating data suggests that blood-based biomarkers have adequate sensitivity and specificity to be advantageous for selecting subjects with AD pathology although they are not yet supported for use as endpoints (25). Incorporating plasma Aβ testing into screening protocols could dramatically reduce the number of PET scans required. Many of the AD platform trials underway or in the planning stages rely on PET scans prior to randomization to determine how far the disease as progressed. For trials testing tau-based therapies, tau PET promises to be especially useful since deposition of neocortical tau is seen even in the presymptomatic stage. PET scans are also commonly used as endpoints and to assess target engagement and treatment response; however, the spectrum of baseline amyloidosis is broad. The growing utility of plasma measures indicative of tau abnormalities will extend biomarker coverage beyond the fibrillar deposits measurable by PET scans. The incorporation of brain donation protocols in DIAN-TU and other trials enables the comparison and confirmation of AD pathology to biomarkers and drug effects on AD pathology.

The heterogeneity of the drugs themselves also increase both the potential benefits and the complexity of platform trials. Multi-arm platforms enable the parallel testing of different approaches and are particularly suited to testing combination therapies. However, the complexity of combination trials is decreased if one drug in a combination approach has been approved for a certain population. While there may be theoretical reasons for pursuing a combination of drugs that target both amyloid and tau, designing a regimen that allows for maximal biological engagement remains unclear, i.e., should amyloid be cleared or neutralized prior to targeting tau, or can targeting tau even in the presence of amyloid stop tau spread and downstream tau effects? Another issue with combination therapies is that two drugs with separate development plans may not be compatible from a logistical standpoint.

Different modes of administration further complicate platform trials. Participants’ acceptance of a treatment may vary by mode of administration (e.g., oral more likely to be accepted than an intrathecal treatment). Combining placebos when there are different modes of administration may not be feasible.

Platform trials may employ different analytical methods to test hypotheses, address subgroup effects, and compare outcomes against control groups (26). Interim analysis offers both benefits and risks. While interim analysis may be informative and can protect subjects from risks of harm or better target limited resources by eliminating a drug that has little chance of success, early decisions may be inaccurate and prematurely ending an arm may also limit what can be learned from the study or reduce confidence in an approach that may ultimately work. Task Force members agreed that planning for interim analysis ahead of time is essential. This includes determining the types of data to be analyzed (e.g. biomarkers vs. cognitive outcomes) and the timepoints at which interim analyses should be conducted. For exploratory, hypothesis-generating platform trials where the goal is to elucidate pathophysiological interactions between targets and drugs, shorter studies with no interim analysis may be appropriate. However, in platform trials with registration as an ultimate goal, carefully designed interim analysis may be appropriate.

Platform studies may also be challenging from a company perspective since they are expensive, complex, and may compete for resources with individual molecule programs. Collaboration of different company sponsors has the potential to share costs and risks, but presents other challenges including protection of intellectual property and data. Industry representatives acknowledged, however, that as data accumulate in a platform and the ability to use that data grows, care must be taken to ensure the data are appropriately used. The Task Force also recognized the importance of ensuring that the community of funders and sponsors understand the benefits and challenges associated with platform trials and that both public and private support are essential for this complex endeavor.

Despite the challenges, many Task Force members believe that platform trials are essential to ensure that the field builds on and moves past the previous disappointments that have plagued amyloid trials. Moreover, they are necessary since conducting trials sequentially, drug by drug and severity stage by severity stage, takes much too long. Platform trials provide other opportunities that are essential for AD drug development by testing the predictive value of biomarker status and the surrogate value of biomarkers; evaluating individual drugs across multiple stages of disease; and accelerating the timing of testing combinations of drugs, which have historically waited until each drug in the combination has shown efficacy. Additionally, the Task Force advocated the importance of continued collaborations and partnerships to ensure that multiple investigators analyze multiple datasets, share data and knowledge, and learn from one another to gain insight into how to defeat this deadly disease.

## References

[1] Rafii MS, Aisen PS (2020). The search for Alzheimer disease therapeutics — same targets, better trials?. Nat Rev Neurol..

[2] Cummings J, Lee G, Ritter A, Sabbagh M, Zhong K (2020). Alzheimer’s disease drug development pipeline: 2020. Alzheimer’s & Dementia: Translational Research & Clinical Interventions.

[3] van Dorn A (2020). COVID-19 and readjusting clinical trials. The Lancet.

[4] Bateman RJ, Benzinger TL, Berry S, Clifford DB, Duggan C, Fagan AM (2017). The DIAN-TU Next Generation Alzheimer’s prevention trial: adaptive design and disease progression model. Alzheimers Dement..

[5] Bateman RJ, Xiong C, Benzinger TLS, Fagan AM, Goate A, Fox NC (2012). Clinical and biomarker changes in dominantly inherited Alzheimer’s disease. N Engl J Med..

[6] Morris JC (1997). Clinical Dementia Rating: A Reliable and Valid Diagnostic and Staging Measure for Dementia of the Alzheimer Type. Int Psychogeriatr..

[7] Strobel G. Topline Result for First DIAN-TU Clinical Trial: Negative on Primary | ALZFORUM [Internet]. ALZFORUM. 2020 [cited 2021 Feb 12]. Available from: https://www.alzforum.org/news/research-news/topline-result-first-dian-tu-clinical-trial-negative-primary

[8] Strobel G. In DIAN-TU, Gantenerumab Brings Down Tau. By a Lot. Open Extension Planned [Internet]. ALZFORUM. 2020 [cited 2021 Feb 12]. Available from: https://www.alzforum.org/news/conference-coverage/dian-tugantenerumab-brings-down-tau-lot-open-extension-planned

[9] Hanseeuw BJ, Betensky RA, Jacobs HIL, Schultz AP, Sepulcre J, Becker JA, et al. Association of Amyloid and Tau With Cognition in Preclinical Alzheimer Disease: A Longitudinal Study. JAMA Neurol. 2019 Jun 3 10.1001/jamaneurol.2019.1424 PMC6547132 31157827

[10] Farrell ME, Jiang S, Schultz AP, Properzi MJ, Price JC, Becker JA, et al. Defining the lowest threshold for amyloid-PET to predict future cognitive decline and amyloid accumulation. Neurology. 2020 Nov 16 10.1212/WNL.0000000000011214 PMC7905788 33199430

[11] Villain N, Chételat G, Grassiot B, Bourgeat P, Jones G, Ellis KA (2012). Regional dynamics of amyloid-β deposition in healthy elderly, mild cognitive impairment and Alzheimer’s disease: a voxelwise PiB-PET longitudinal study. Brain.

[12] Jack CR, Therneau TM, Wiste HJ, Weigand SD, Knopman DS, Lowe VJ (2016). Transition rates between amyloid and neurodegeneration biomarker states and to dementia: a population-based, longitudinal cohort study. Lancet Neurol..

[13] Cummings J, Blennow K, Johnson K, Keeley M, Bateman RJ, Molinuevo JL (2019). Anti-Tau Trials for Alzheimer’s Disease: A Report from the EU/US/CTAD Task Force. J Prev Alzheimers Dis..

[14] Barthélemy NR, Li Y, Joseph-Mathurin N, Gordon BA, Hassenstab J, Benzinger T (2020). A soluble phosphorylated tau signature links tau, amyloid and the evolution of stages of dominantly inherited Alzheimer’s disease. Nat Med..

[15] Mullard A. Failure of first anti-tau antibody in Alzheimer disease highlights risks of history repeating. Nature Reviews Drug Discovery [Internet]. 2020 Dec 10 [cited 2020 Dec 10]; Available from: https://www.nature.com/articles/d41573-020-00217-7 10.1038/d41573-020-00217-7 33303932

[16] Woodcock J, LaVange LM (2017). Master Protocols to Study Multiple Therapies, Multiple Diseases, or Both. N Engl J Med..

[17] (2021). Sci Transl Med..

[18] Ritchie CW, Muniz-Terrera G, Kivipelto M, Solomon A, Tom B, Molinuevo JL (2020). The European Prevention of Alzheimer’s Dementia (EPAD) Longitudinal Cohort Study: Baseline Data Release V500.0. J Prev Alzheimers Dis..

[19] Rosenberg A, Mangialasche F, Ngandu T, Solomon A, Kivipelto M (2020). Multidomain Interventions to Prevent Cognitive Impairment, Alzheimer’s Disease, and Dementia: From FINGER to World-Wide FINGERS. J Prev Alzheimers Dis..

[20] Aisen PS, Sperling RA, Cummings J, Donohue MC, Langford O, Jimenez-Maggiora GA (2020). The Trial-Ready Cohort for Preclinical/Prodromal Alzheimer’s Disease (TRC-PAD) Project: An Overview. J Prev Alzheimers Dis..

[21] Walter S, Clanton TB, Langford OG, Rafii MS, Shaffer EJ, Grill JD (2020). Recruitment into the Alzheimer Prevention Trials (APT) Webstudy for a Trial-Ready Cohort for Preclinical and Prodromal Alzheimer’s Disease (TRC-PAD). J Prev Alzheimers Dis..

[22] Walter S, Langford OG, Clanton TB, Jimenez-Maggiora GA, Raman R, Rafii MS (2020). The Trial-Ready Cohort for Preclinical and Prodromal Alzheimer’s Disease (TRC-PAD): Experience from the First 3 Years. J Prev Alzheimers Dis..

[23] Jimenez-Maggiora GA, Bruschi S, Raman R, Langford O, Donohue M, Rafii MS (2020). TRC-PAD: Accelerating Recruitment of AD Clinical Trials through Innovative Information Technology. J Prev Alzheimers Dis..

[24] Langford O, Raman R, Sperling RA, Cummings J, Sun C-K, Jimenez-Maggiora G (2020). Predicting Amyloid Burden to Accelerate Recruitment of Secondary Prevention Clinical Trials. J Prev Alzheimers Dis..

[25] Palmqvist S, Janelidze S, Quiroz YT, Zetterberg H, Lopera F, Stomrud E (2020). Discriminative Accuracy of Plasma Phospho-tau217 for Alzheimer Disease vs Other Neurodegenerative Disorders. JAMA.

[26] Park JJH, Harari O, Dron L, Lester RT, Thorlund K, Mills EJ (2020). An overview of platform trials with a checklist for clinical readers. Journal of Clinical Epidemiology.

